# Explainable machine learning predicts survival of retroperitoneal liposarcoma: A study based on the SEER database and external validation in China

**DOI:** 10.1002/cam4.7324

**Published:** 2024-06-07

**Authors:** Maoyu Wang, Zhizhou Li, Shuxiong Zeng, Ziwei Wang, Yidie Ying, Wei He, Zhensheng Zhang, Huiqing Wang, Chuanliang Xu

**Affiliations:** ^1^ Department of Urology Shanghai Changhai Hospital, Naval Medical University Shanghai China

**Keywords:** explainable machine learning, overall survival, retroperitoneal liposarcoma, SurvLIME, SurvSHAP

## Abstract

**Objective:**

We have developed explainable machine learning models to predict the overall survival (OS) of retroperitoneal liposarcoma (RLPS) patients. This approach aims to enhance the explainability and transparency of our modeling results.

**Methods:**

We collected clinicopathological information of RLPS patients from The Surveillance, Epidemiology, and End Results (SEER) database and allocated them into training and validation sets with a 7:3 ratio. Simultaneously, we obtained an external validation cohort from The First Affiliated Hospital of Naval Medical University (Shanghai, China). We performed LASSO regression and multivariate Cox proportional hazards analysis to identify relevant risk factors, which were then combined to develop six machine learning (ML) models: Cox proportional hazards model (Coxph), random survival forest (RSF), ranger, gradient boosting with component‐wise linear models (GBM), decision trees, and boosting trees. The predictive performance of these ML models was evaluated using the concordance index (C‐index), the integrated cumulative/dynamic area under the curve (AUC), and the integrated Brier score, as well as the Cox–Snell residual plot. We also used time‐dependent variable importance, analysis of partial dependence survival plots, and the generation of aggregated survival SHapley Additive exPlanations (SurvSHAP) plots to provide a global explanation of the optimal model. Additionally, SurvSHAP (t) and survival local interpretable model‐agnostic explanations (SurvLIME) plots were used to provide a local explanation of the optimal model.

**Results:**

The final ML models are consisted of six factors: patient's age, gender, marital status, surgical history, as well as tumor's histopathological classification, histological grade, and SEER stage. Our prognostic model exhibits significant discriminative ability, particularly with the ranger model performing optimally. In the training set, validation set, and external validation set, the AUC for 1, 3, and 5 year OS are all above 0.83, and the integrated Brier scores are consistently below 0.15. The explainability analysis of the ranger model also indicates that histological grade, histopathological classification, and age are the most influential factors in predicting OS.

**Conclusions:**

The ranger ML prognostic model exhibits optimal performance and can be utilized to predict the OS of RLPS patients, offering valuable and crucial references for clinical physicians to make informed decisions in advance.

## 1 INTRODUCTION

Retroperitoneal liposarcoma (RLPS) commonly presents as large mass lesions discovered incidentally on imaging studies conducted to evaluate unrelated symptoms, typically appearing at a later stage.^1^ Retroperitoneal sarcoma (RPS) is a rare malignant neoplasm, with an approximate incidence of 0.5–1 per 100,000 individuals.^2^ RLPS represents the predominant pathological subtype of RPS, accounting for approximately 45% of cases.^3^ Pathologically, RLPS is classified into five distinct categories: dedifferentiated liposarcoma (DDLPS), well‐differentiated liposarcoma (WDLPS), pleomorphic liposarcoma (PLS), myxoid liposarcoma (MLS), and myxoid pleomorphic liposarcoma.^4^ WDLPS typically exhibits a relatively indolent malignant behavior, although it may display local aggressiveness. In contrast, DDLPS demonstrates a higher histological grade, rapid growth, and the potential for distant metastasis.^5^ Currently, complete resection represents the sole approach to achieve a radical cure. Given the lack of discernible clinical benefits in adjuvant therapies, including chemotherapy, radiotherapy, and targeted therapy, for RLPS, surgical intervention remains the primary therapeutic modality.^6^, ^7^, ^8^ However, a previous study involving a substantial cohort of patients who underwent complete resections demonstrated a 54% overall survival (OS) rate among RLPS patients over a 5‐year period.^9^ Currently, improving the survival rate of RLPS patients has become a challenge confronted by clinicians.

Machine learning (ML) possesses the capability to leverage the most recent advancements in large datasets to enhance health care delivery with increased efficiency and precision through automated identification and learning of patterns from data.^10^ Recently, it has emerged as a highly promising technology that employs statistical techniques to deduce correlations between patient characteristics and outcomes, enabling objective data integration for outcome prediction.^11^ Compared with conventional regression methods, ML algorithms possess superior performance in predicting outcomes within vast databases.^12^ In addition, the explainable machine learning algorithms can assist in transforming opaque “black box” predictions yielded by ML into more comprehensible “white box” interpretations.^13^ Zhang et al. established a random forest model using machine learning to accurately predict the risk in patients with non‐squamous cell carcinoma of the head and neck.^14^ By evaluating three distinct ML algorithms, Fan et al. ultimately formulated a CoxBoost model with the best performance for prognosticating survival in patients with spinal and pelvic Ewing's sarcoma.^15^

Therefore, our study aims to construct six models for survival analysis and evaluate their performance using diverse operational methods, with the objective of identifying an accurate predictive model that can effectively guide the selection of clinical diagnosis and treatment strategies. The data utilized in our models were obtained from The Surveillance, Epidemiology, and End Results (SEER) database of the National Cancer Institute. Additionally, we externally validated the model using an independent dataset from The First Affiliated Hospital of Naval Medical University (Shanghai, China).

## 2 MATERIALS AND METHODS

### 2.1 Data acquisition

**FIGURE 1:**
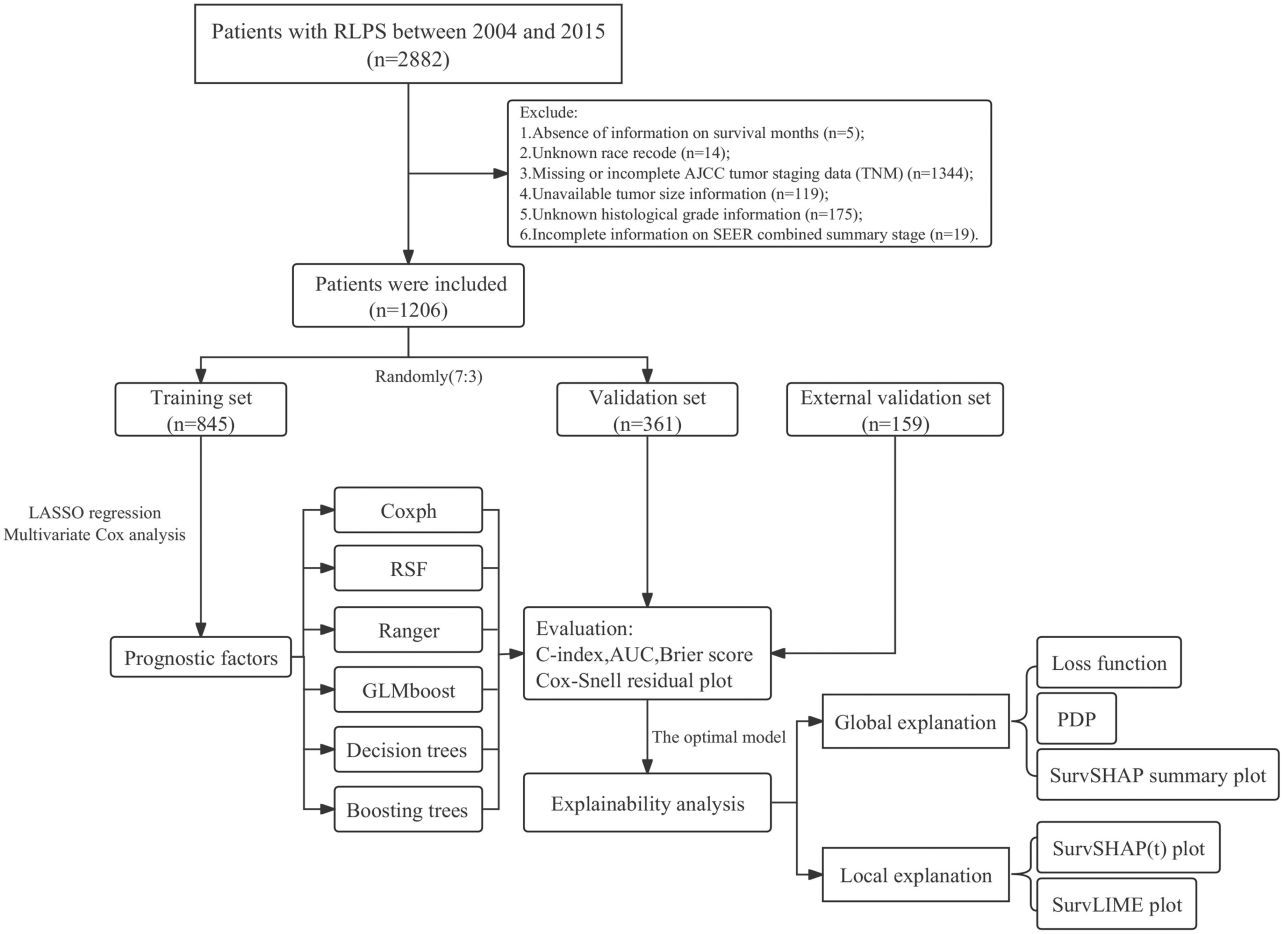
Work flow chart.

In this study, we conducted an analysis of clinical data obtained from patients diagnosed with RLPS between 2004 and 2015. The patient records were identified using the International Classification of Diseases for Oncology, third edition (ICD‐O‐3) codes (8850–8858) from SEER program. Access to the SEER research data was facilitated using SEER*Stat 8.4.1.2 software, which was downloaded from the official website (http://seer.cancer.gov//seerstat/). Exclusion criteria were implemented to ensure data quality, and included the following: (1) absence of information on survival months; (2) unknown race information; (3) missing or incomplete American Joint Committee on Cancer (AJCC) tumor staging data (T stage, N stage, and M stage); (4) unavailable tumor size information; (5) unknown histological grade information; (6) incomplete information on SEER combined summary stage. The screening process of the SEER database is illustrated in Figure 1.

According to the specified inclusion and exclusion criteria, a total of 1206 patients were recruited for this study. These patients were then randomly allocated into two groups: the training set (*n* = 845) and the validation set (*n* = 361), maintaining a 7:3 ratio. In addition, the clinical data for the external validation set (*n* = 159) were obtained from patients treated at The First Affiliated Hospital of Naval Medical University between 2001 and 2019. Throughout the duration of the study, data collection was conducted by three independent investigators. Two investigators extracted the data, while a third investigator ensured its accuracy. It is important to note that all patient data have been anonymized, eliminating the need for informed consent.

### 2.2 Variable collection

This study collected various clinical parameters, including age at diagnosis, gender, race, marital status, tumor size, histological grade, SEER stage, AJCC stage, AJCC 7th TNM stage, histopathological classification, and treatment modality (surgery and radiation). The primary outcome measure employed in this research was OS, which was defined as the time interval between the date of diagnosis and either the occurrence of death or the most recent follow‐up. To determine the optimal cutoff points for age and tumor size, X‐Tile software (version 3.6.1) was utilized.^16^ Age was dichotomized into two groups using a cutoff of 65 years old, whereas tumor size was categorized based on cutoffs of 10 cm and 20 cm. Moreover, RLPS histology was classified according to the WHO system into WDLPS, MLS, PLS, DDLPS, and other subtypes (round cell, angiomyoliposarcoma, mixed, and not otherwise specified). Race was classified into three categories: White, Black, or Other (including Asian or Pacific Islander or American Indian/Alaska Native), whereas marital status was categorized as married or other (divorced, widowed, single, separated, or unknown).

### 2.3 Identification of prognostic factors for survival

All variables in this study were transformed into categorical variables and presented as frequencies and proportions. To mitigate overfitting, the least absolute shrinkage and selection operator (LASSO) method was utilized to primarily select relevant predictive features. Subsequently, significant prognostic factors were identified through multivariate analysis using the Cox proportional hazards model. The corresponding 95% confidence interval (CI) was calculated for all potential risk factors.

### 2.4 Model developments

In this study, five advanced machine learning algorithms were employed to formulate prognostic models. Each algorithm possesses distinct capabilities that surpass the traditional Cox proportional hazard (Coxph) model.

The random survival forest (RSF) method integrates random forest with survival analysis techniques to handle right‐censored data. It introduces novel survival splitting rules for the growth of survival trees and innovative algorithms for estimating missing data. Moreover, RSF incorporates the event retention principle of survival forests, using it to define overall mortality rates—a simple and interpretable measure that can serve as a predictive outcome.^17^, ^18^ RSF computation can be executed via the R‐package randomForestSRC (rfsrc).^19^

Ranger is typically a decision tree‐based method used for addressing classification and regression problems. Notably, it demonstrates swift implementation through random forests or recursive partitioning, making it especially advantageous for high‐dimensional datasets.^20^ The model undergoes extensive training involving a multitude of survival trees, and the ultimate prediction is derived through a weighted voting system based on the contributions of individual trees.^21^, ^22^

Gradient boosting with component‐wise linear models (GLMboost) is an algorithm for regression and classification, based on gradient boosting trees, with the generalized linear model (GLM) serving as its base model.^23^ Using a progressive gradient boosting approach, GLMBoost systematically improves the predictive capability of the underlying model while simultaneously simplifying the model complexity through regularization. One unique advantage of GLMBoost is its adaptability to a wide range of data types, including both categorical and continuous variables. Additionally, it demonstrates the capability to address the common challenge of missing data, which is often encountered in datasets representing real‐world scenarios.^24^

Decision trees predict the values of the dependent variable by acquiring simple decision rules derived from the available data.^25^, ^26^ In contrast, boosting trees are an ensemble learning method that focuses on combining predictions from multiple models to improve overall performance.^27^ Decision trees prioritize increasing model accuracy by selecting the most suitable feature for partitioning the data, whereas boosting trees combine multiple models to achieve superior generalization performance.

### 2.5 Model tests and evaluation

The performance of six different models was assessed using various metrics, including the concordance index (C‐index), the integrated cumulative/dynamic area under the curve (AUC), and the integrated Brier score, as well as the Cox–Snell residual plot. The C‐index measures the discriminatory ability of the survival model, indicating practical utility when its value is above 0.7. An increased likelihood of successful model prediction is associated with a higher C‐index.^28^ In a similar manner, the AUC calculated from the ROC curve serves as a comparable measure to the C‐index. Furthermore, the Brier score is utilized to assess the predictive accuracy of the model, with values below 0.25 suggesting practical application. Improved predictive accuracy is associated with lower Brier scores. To evaluate the performance of the six prediction models more visually, curves were generated to illustrate the changes in C‐index and Brier score values over time. Moreover, the Cox–Snell residual plot is a graphical tool used to assess the goodness of fit of a model.^29^

### 2.6 Model interpretability

The optimal utilization of artificial intelligence in medicine highlights the necessity for intuitive interpretations of black box ML models and subsequent confirmations of their practical significance. To address this, a dual approach involving both global and local explanations is employed for models demonstrating superior fittings. Initially, a global explanation was conducted by the entire cohort, using assessments such as time‐dependent variable importance, analysis of partial dependence survival plots, and the generation of aggregated survival SHapley Additive exPlanations (SurvSHAP) plots.^30^, ^31^ Subsequently, the focus shifted to localized explanations for individual statistical units, specifically single patients, derived from the examination of SurvSHAP (t) and survival local interpretable model‐agnostic explanations (SurvLIME) plots.^32^

### 2.7 Statistical methods

The statistical analyses were conducted using the R version 4.2.1 software, which leveraged various packages such as “survex,” “parsnip,” “mlr3proba,” “ranger,” “randomForestSRC,” and “survival.” To detect differences between variable sets, the chi‐squared test or Fisher exact test was employed. A significance level of *p* < 0.05 was considered statistically significant.

## 3 RESULTS

### 3.1 Demographic and clinicopathological characteristics

Using predetermined inclusion and exclusion criteria, a comprehensive cohort of 1206 patients diagnosed with RLPS between 2004 and 2015 were meticulously chosen from the SEER database. Among them, 845 patients were allocated to the training set, while the remaining 361 patients constituted the validation set. The application of the chi‐squared test for statistical analysis showed no significant disparity between the training and validation sets. The clinicopathological characteristics of both sets are delineated in Table 1. In the SEER database, the majority of patient with RLPS were of White race (84.25%), predominantly male (54.56%), primarily below the age of 65 years (53.65%), and commonly married (65.42%). Moreover, a significant proportion of cases (47.84%) involved tumors exceeding 20 cm in size. SEER staging categorizes cases into local, regional, and distant stages, with local staging representing the highest proportion (49.75%). Using the 7th TNM stage of AJCC, the distribution reveals that a majority fell into T2 (95.61%), N0 (95.77%), and M0 (95.94%), respectively, with Stage I/II (58.54%) being the most prevalent in AJCC staging. The study includes patients classified pathologically as DDLS (38.81%), WDLS (36.57%), MLS (4.73%), PLS (1.99%), and other liposarcoma subtypes (17.91%), with a higher occurrence of Grade I/II (59.29%) in histologic grading. Concerning treatment modalities, nearly all patients underwent surgery (93.70%), while a minority received radiotherapy (25.04%). Table S1 presents the clinicopathological characteristics of patients selected from Changhai hospital. Most patients with RLPS were male, accounting for 57.86% of the total, and they were predominantly below the age of 65, at 63.52%. Additionally, most of them were married, constituting 81.13% of the population. Furthermore, SEER staging, with regional staging being the most common, accounts for 52.83% of the cases. The study encompasses patients who have been pathologically classified as DDLS (45.91%), WDLS (22.01%), MLS (7.55%), PLS (4.40%), and other liposarcoma subtypes (20.13%). Histologic grading reveals a higher occurrence of Grade III/IV, accounting for 54.72% of the cases. Regarding treatment modalities, almost all patients underwent surgery, which constituted 92.45% of the total number of patients.

**TABLE 1:** Clinicopathological characteristics of RLPS in SEER database.

	Total (*n* = 1206)		Training set (*n* = 845)		Validation set (*n* = 361)		*p*‐value^a^
	No. of patients	%	No. of patients	%	No. of patients	%	
*Age, years*							
<65	647	0.54	451	0.53	196	0.54	0.818
≥65	559	0.46	394	0.47	165	0.46	
*Sex*							
Female	548	0.45	381	0.45	167	0.46	0.756
Male	658	0.55	464	0.55	194	0.54	
*Race*							
White	1016	0.84	714	0.84	302	0.84	0.403
Black	64	0.05	48	0.06	16	0.04	
Other	126	0.10	83	0.10	43	0.12	
*Marital status*							
Married	789	0.65	551	0.65	238	0.66	0.861
Other	417	0.35	294	0.35	123	0.34	
*Tumor size*							
<10	202	0.17	140	0.17	62	0.17	0.895
10–20	427	0.35	297	0.35	130	0.36	
>20	577	0.48	408	0.48	169	0.47	
*Histological grade* ^b^							
I/II	715	0.59	503	0.60	212	0.59	0.845
III/IV	491	0.41	342	0.40	149	0.41	
*SEER stage*							
Localized	600	0.50	425	0.50	175	0.48	0.746
Regional	499	0.41	348	0.41	151	0.42	
Distant	107	0.09	72	0.09	35	0.10	
*AJCC stage*							
I/II	706	0.59	498	0.59	208	0.58	0.718
III/IV	500	0.41	347	0.41	153	0.42	
*AJCC T*							
T1	53	0.04	32	0.04	21	0.06	0.155
T2	1153	0.96	813	0.96	340	0.94	
*AJCC N*							
N0	1155	0.96	808	0.96	347	0.96	0.818
N1	19	0.02	13	0.02	6	0.02	
NX	32	0.03	24	0.03	8	0.02	
*AJCC M*							
M0	1157	0.96	812	0.96	345	0.96	0.791
M1	49	0.04	33	0.04	16	0.04	
*Histopathological classification*							
DDLPS	468	0.39	325	0.38	143	0.40	0.851
MLS	57	0.05	37	0.04	20	0.06	
PLS	24	0.02	18	0.02	6	0.02	
WDLPS	441	0.37	314	0.37	127	0.35	
Other	216	0.18	151	0.18	65	0.18	
*Surgery*							
Yes	1130	0.94	797	0.94	333	0.92	0.219
None/unknown	76	0.06	48	0.06	28	0.08	
*Radiotherapy*							
Yes	302	0.25	214	0.25	88	0.24	0.783
None/unknown	904	0.75	631	0.75	273	0.76	

^a^ Chi‐squared test.

^b^ Grade I: well differentiated; Grade II: moderately differentiated; Grade III: poorly differentiated; Grade IV: undifferentiated.

### 3.2 Identification of prognostic factors for retroperitoneal liposarcoma

**FIGURE 2:**
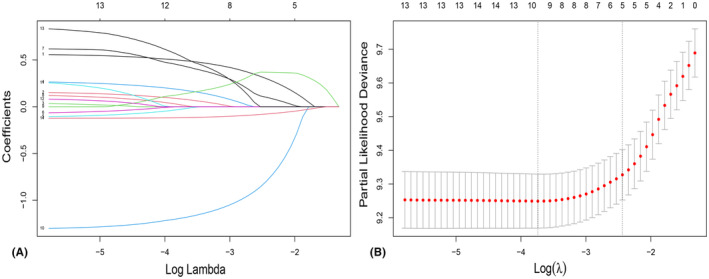
Feature selection using LASSO regression. (A) The LASSO coefficient profiles depict the representation of 14 variables. (B) The selection of tuning parameter (λ) in LASSO regression analysis is determined through 10‐fold cross‐validation. Dotted vertical lines are plotted at the optimal values based on both minimum criteria (right) and “one standard error” criteria (left).

**FIGURE 3:**
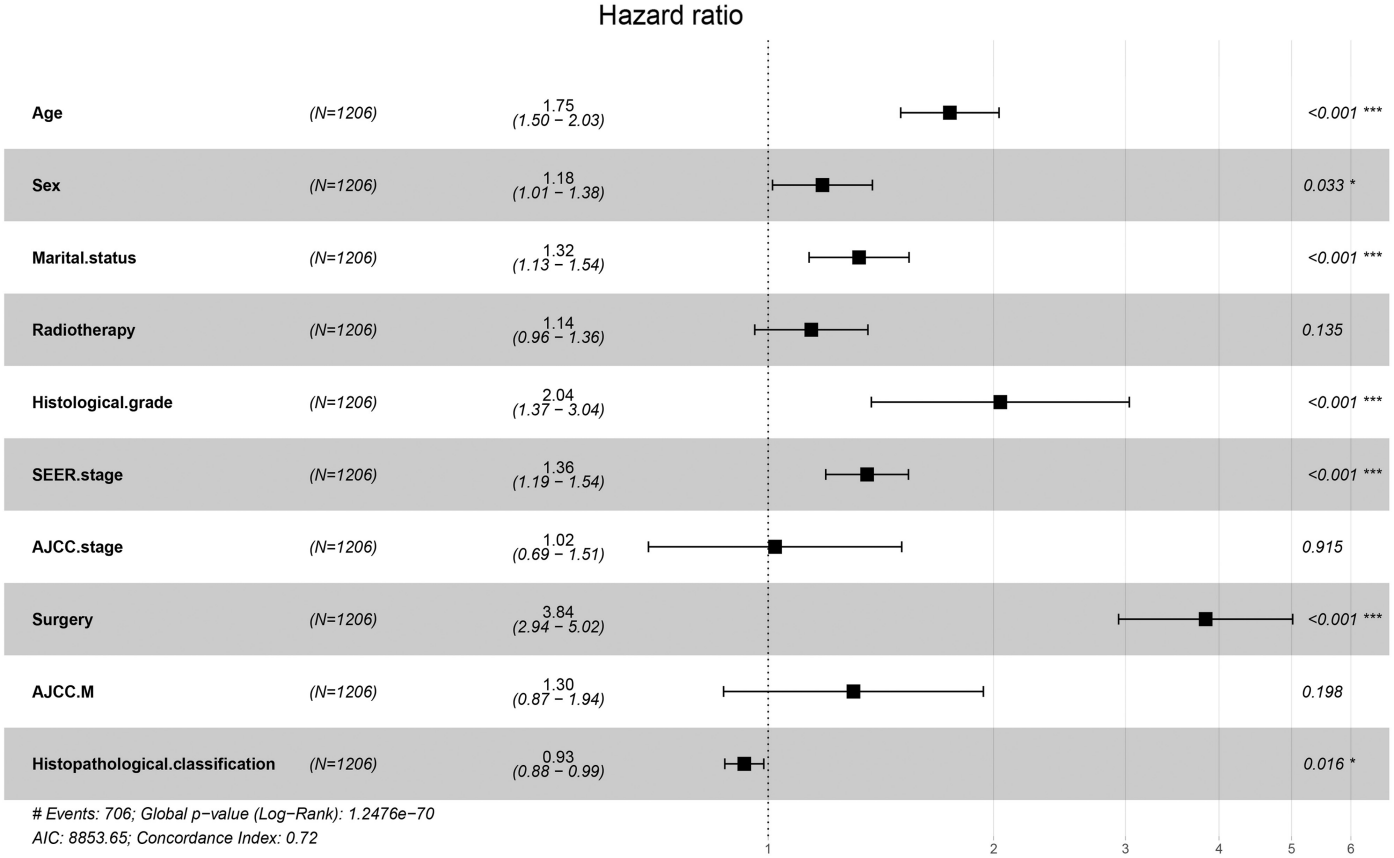
Multivariate analysis of the RLPS in the SEER cohort with 10 variables was conducted.

A total of 14 clinical parameters were incorporated in the SEER set. The LASSO regression, augmented by tenfold cross‐validation, was employed to ascertain predictive factors. The optimal tuning parameter λ for the LASSO regression was determined to be 0.0237, when the partial likelihood binomial deviance reached its lowest point (Figure 2). Ten variables with nonzero coefficients were retained in the LASSO analysis (Figure 2). These ten variables were then integrated into the multivariate Cox analysis (Figure 3). Among these variables, those with a p value below 0.05 were identified as independent risk determinants for OS, including age (HR 1.75, 95%CI: 1.50–2.03) at diagnosis, sex (HR 1.18, 95%CI: 1.01–1.38), marital status (HR 1.32, 95%CI: 1.13–1.54), histopathological classification (HR 0.93, 95%CI: 0.88–0.99), histological grade (HR 2.04, 95%CI: 1.37–3.04), SEER stage (HR 1.36, 95%CI: 1.19–1.54), and surgery (HR 3.84, 95%CI: 2.94–5.02).

### 3.3 Model comparisons

**FIGURE 4:**
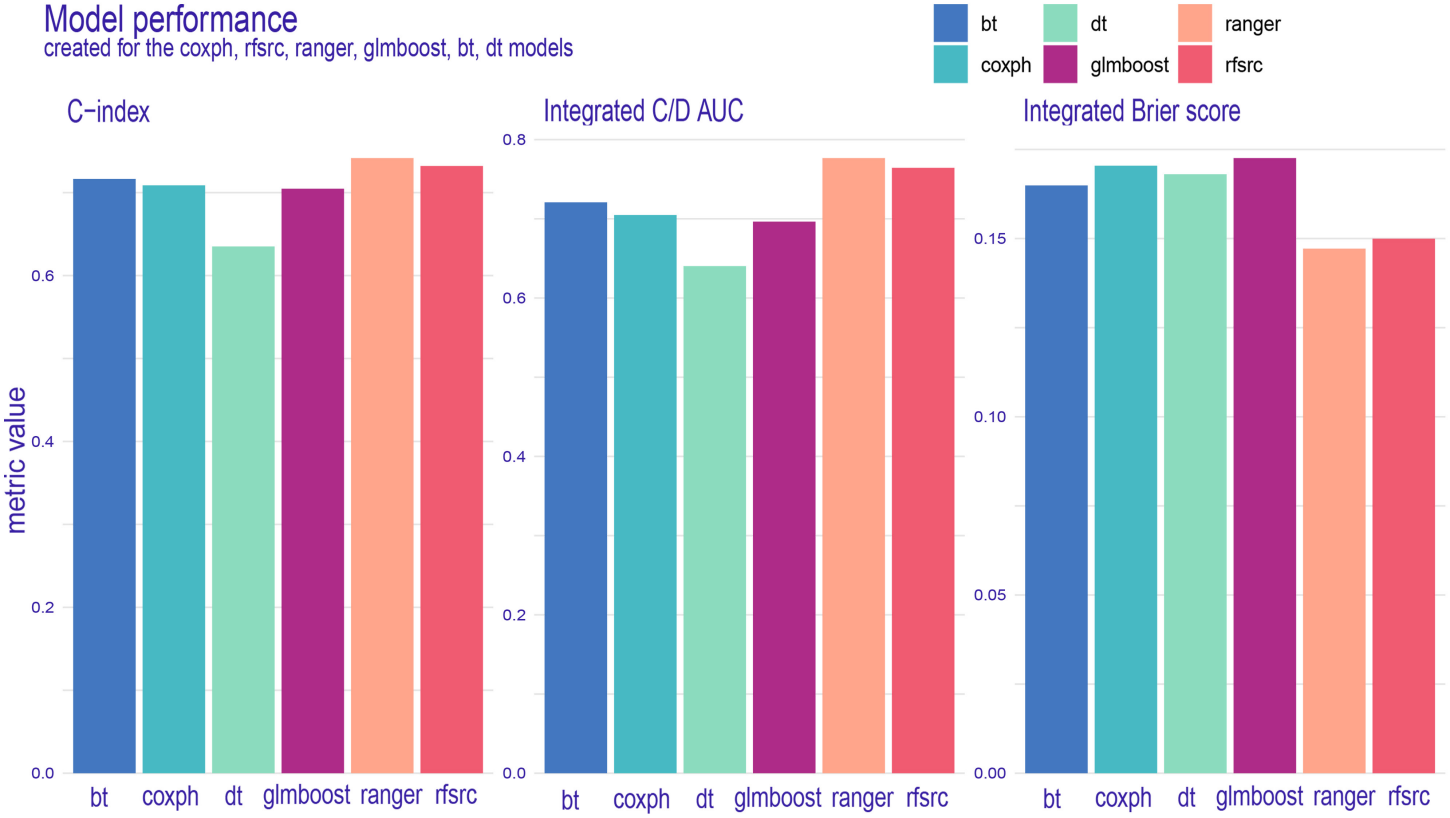
Model performance for the training set was displayed in the form of bar plots. bt, boosting trees; Coxph, Cox proportional hazard; dt, decision trees; glmboost, gradient boosting with component‐wise linear models; rfsrc, random survival forest.

**FIGURE 5:**
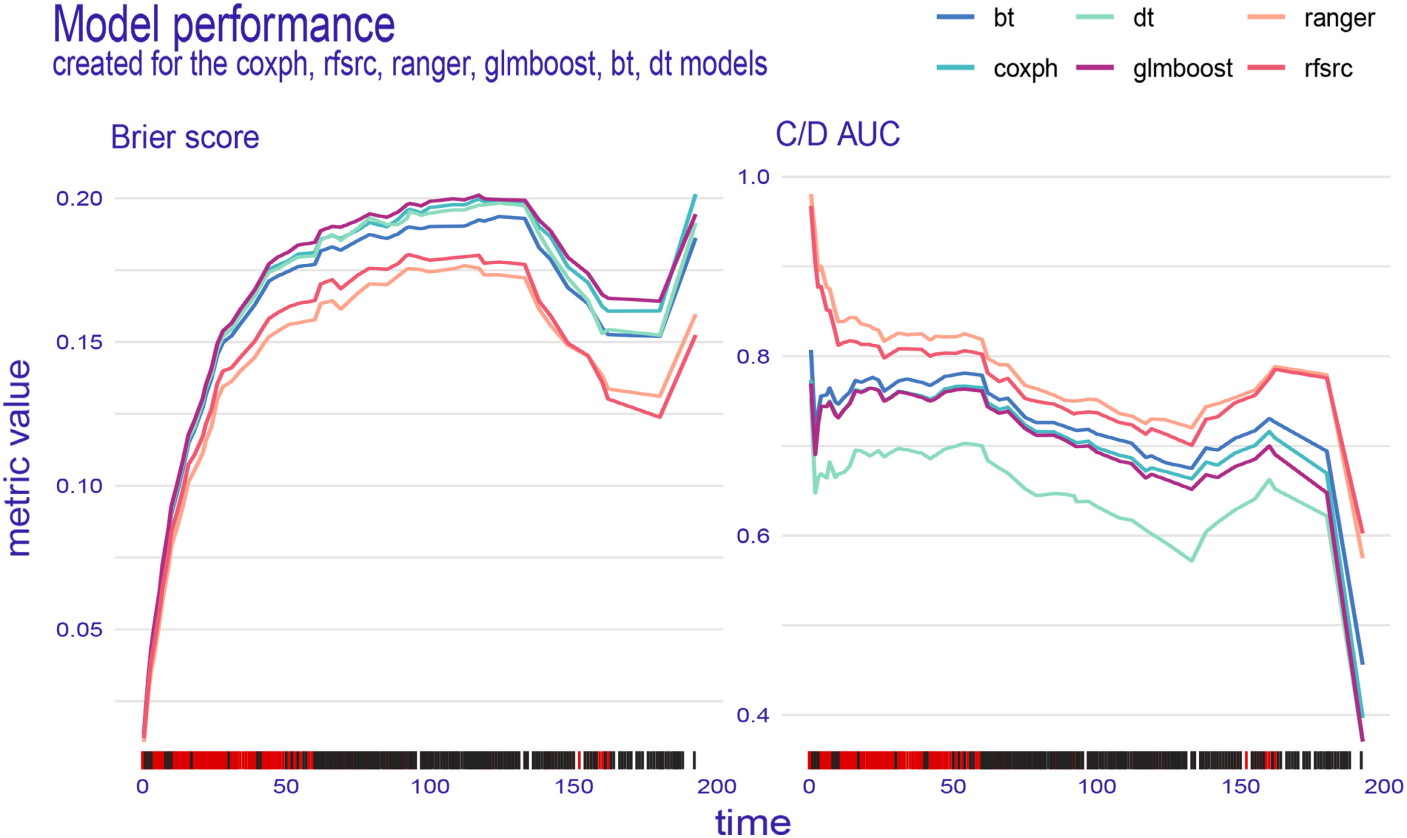
Model performance for the training set was displayed as a line chart that changes over time.

**FIGURE 6:**
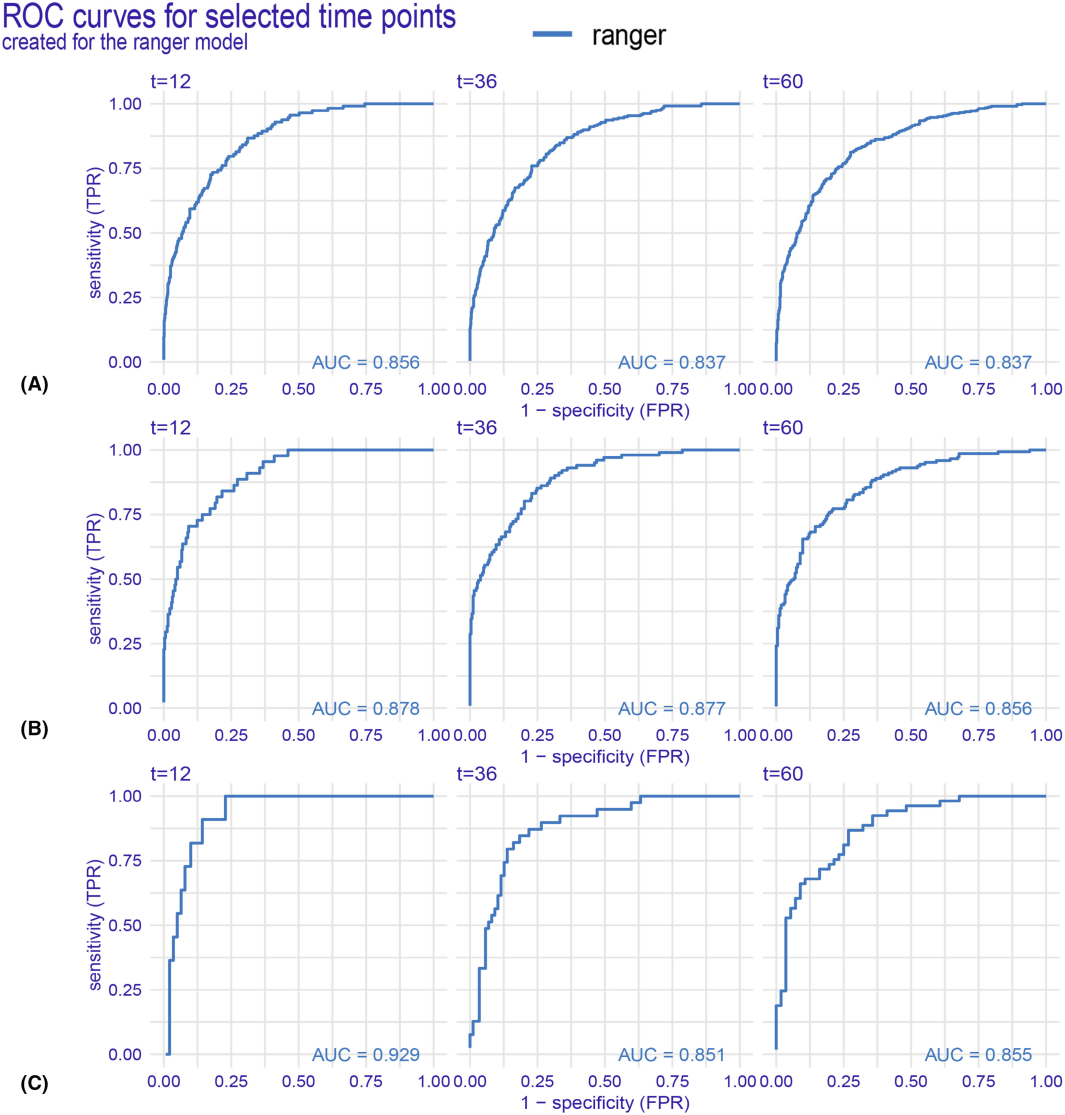
ROC curve analysis of the ranger model was used to evaluate the accuracy of the 1‐, 3‐, and 5‐year predictions. (A) ROC curve for the training set. (B) ROC curve for the validation set. (C) ROC curve for the external validation set.

**FIGURE 7:**
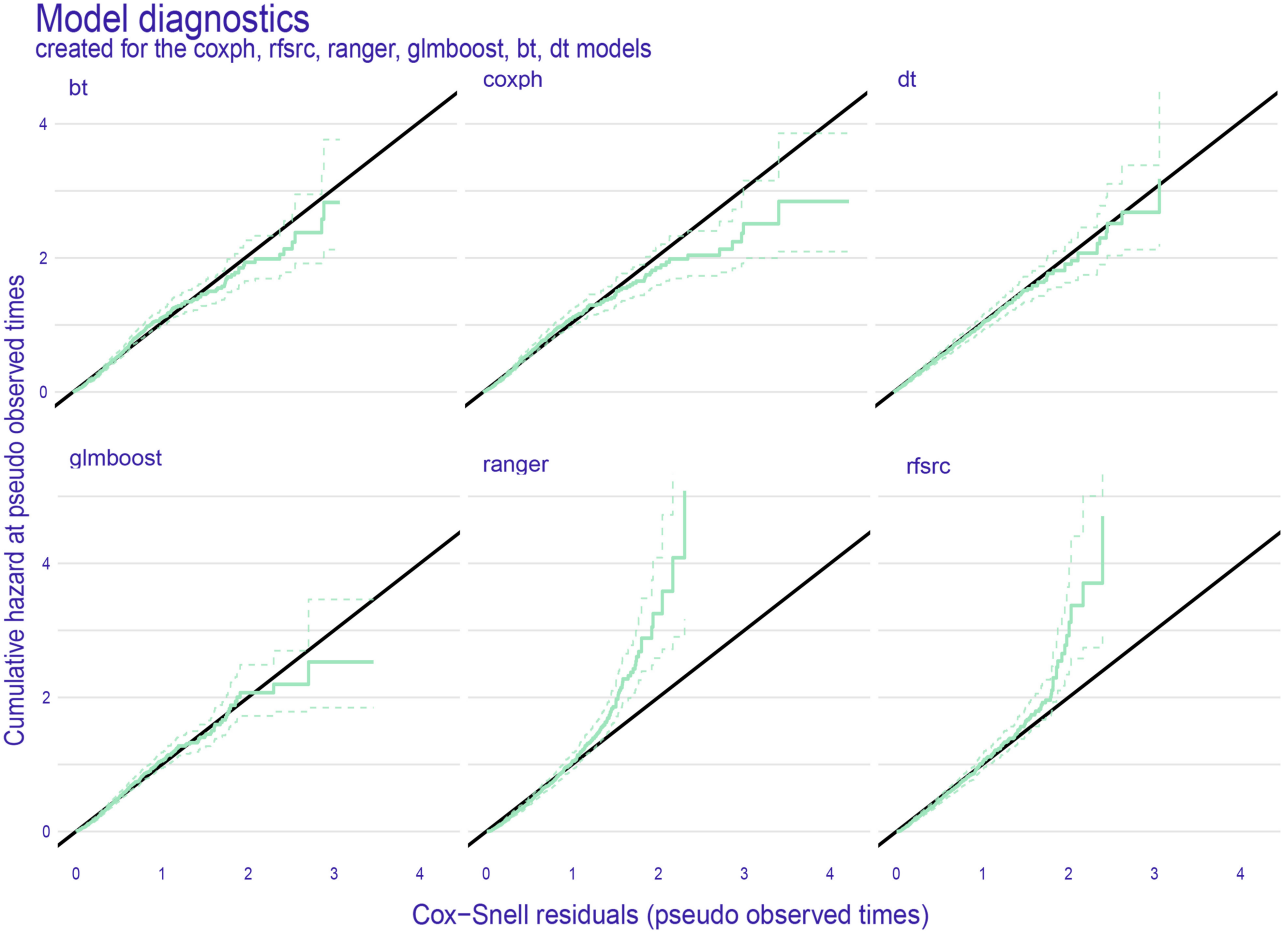
The Cox–Snell residual plots were displayed for all models in the training dataset.

We have developed six machine learning predictive models and utilized both internal and external validation cohorts to evaluate their performance. The results of all models are presented in Table 2, demonstrating remarkable calibration, as evidenced by the integrated Brier score below 0.18 for each model. Notably, the ranger model exhibited superior performance across all metrics (Figure 4) and throughout the entire follow‐up period (Figure 5) in the training dataset. The integrated Brier score, integrated C/D AUC, and C‐index for the ranger model in the training set, validation set, and external validation set were as follows: 0.147, 0.129, and 0.123; 0.777, 0.849, and 0.710; and 0.742, 0.775, and 0.756, respectively. Figures S1–S4 showcase the model performance in each metric and during the entire follow‐up for the validation set and external validation set. The ROC curves (Figure 6) of the ranger model illustrate that the area under the receiver operating characteristic values at 1, 3, and 5 years were as follows: 0.856, 0.837, and 0.837 in the training set; 0.878, 0.877, and 0.856 in the validation set; and 0.929, 0.851, and 0.855 in the external validation set, respectively. Furthermore, the Cox–Snell residual plots (Figure 7) for all models in the training dataset showcased a linear pattern originating from the origin, with a slope closely approximating 1. This observation suggests that our models possess a strong goodness of fit. The Cox–Snell residual plots for the validation set and external validation set can be found in Figures S5 and S6.

**TABLE 2:** The all models' performance in the training set, validation set, and external validation set.

	C‐index	Integrated C/D AUC	Integrated brier score
*Training set*			
Coxph	0.709	0.705	0.170
RSF	0.732	0.764	0.150
Ranger	0.742	0.777	0.147
GLMboost	0.705	0.696	0.173
Boosting trees	0.717	0.721	0.165
Decision trees	0.635	0.640	0.168
*Validation set*			
Coxph	0.717	0.753	0.160
RSF	0.756	0.817	0.139
Ranger	0.775	0.849	0.129
GLMboost	0.711	0.748	0.162
Boosting trees	0.738	0.781	0.148
Decision trees	0.594	0.657	0.154
*External validation set*			
Coxph	0.680	0.688	0.136
RSF	0.705	0.705	0.124
Ranger	0.756	0.710	0.123
GLMboost	0.673	0.672	0.137
Boosting trees	0.629	0.631	0.127
Decision trees	0.303	0.351	0.147

### 3.4 Global explanation

In order to further evaluate the optimal model, we conducted global and local interpretations, aiming to gain a comprehensive understanding of the model's performance.

#### 3.4.1 Time‐dependent feature importance

**FIGURE 8:**
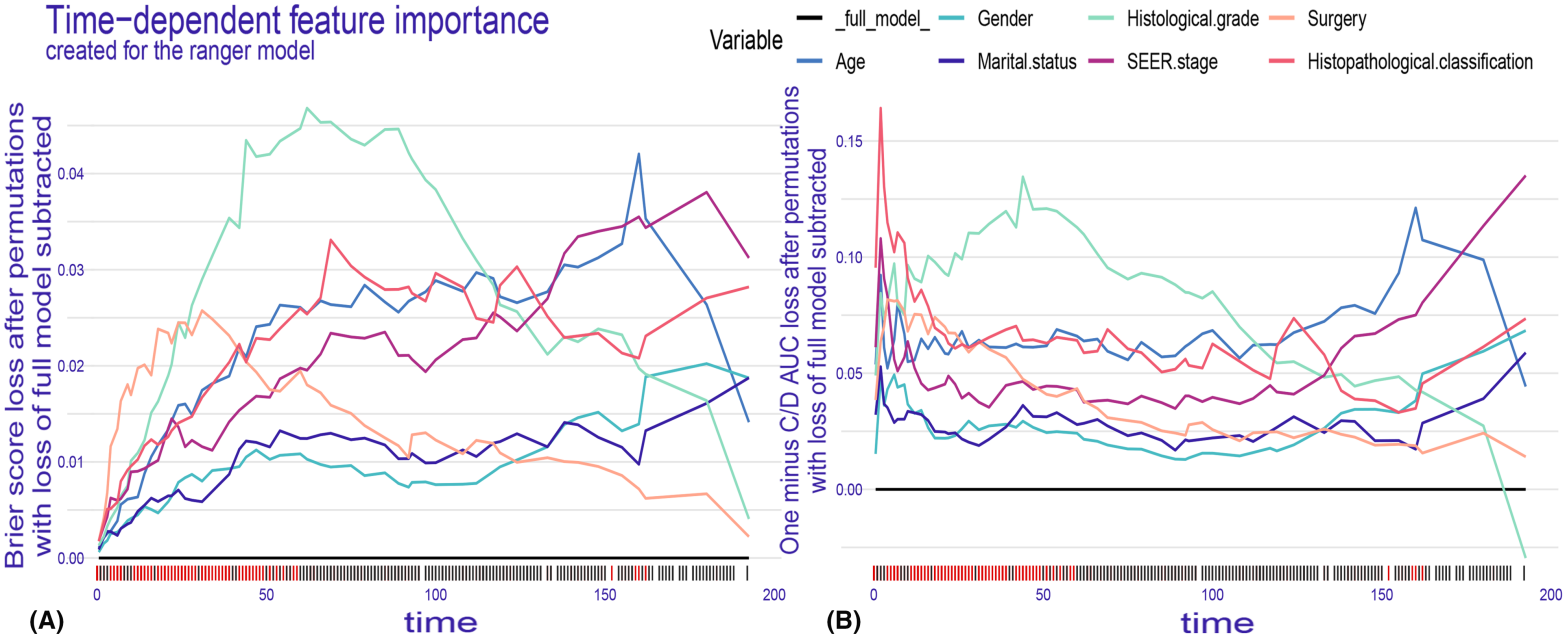
Time‐dependent feature importance for the training set, (A) The Brier score loss after permutation and (B) the C/D AUC loss after permutation. The *y*‐axis represents the variation in the loss function after permuting each covariate.

In our study, we examined how each variable affects the model's predictions on a global scale. Given that each variable may have varying impacts at different time points, we utilized the loss function to compute and rank the importance of the ranger model's variables in the training dataset. Time‐dependent variable importance was investigated using two distinct approaches: the Brier score loss after permutation and the C/D AUC loss after permutation (Figure 8). The importance of variables did indeed change over time, with the highest increase in the loss function being correlated with the most important determinant of OS. The Brier score loss exhibited a more pronounced time‐dependent effect. Our results indicated that, when the survival time was approximately less than 125 months, histological grade was the worst independent hazard factor for OS. Conversely, when the survival time was approximately greater than 125 months, age and SEER stage were the worst independent hazard factors for OS.

#### 3.4.2 Partial dependence survival profiles

**FIGURE 9:**
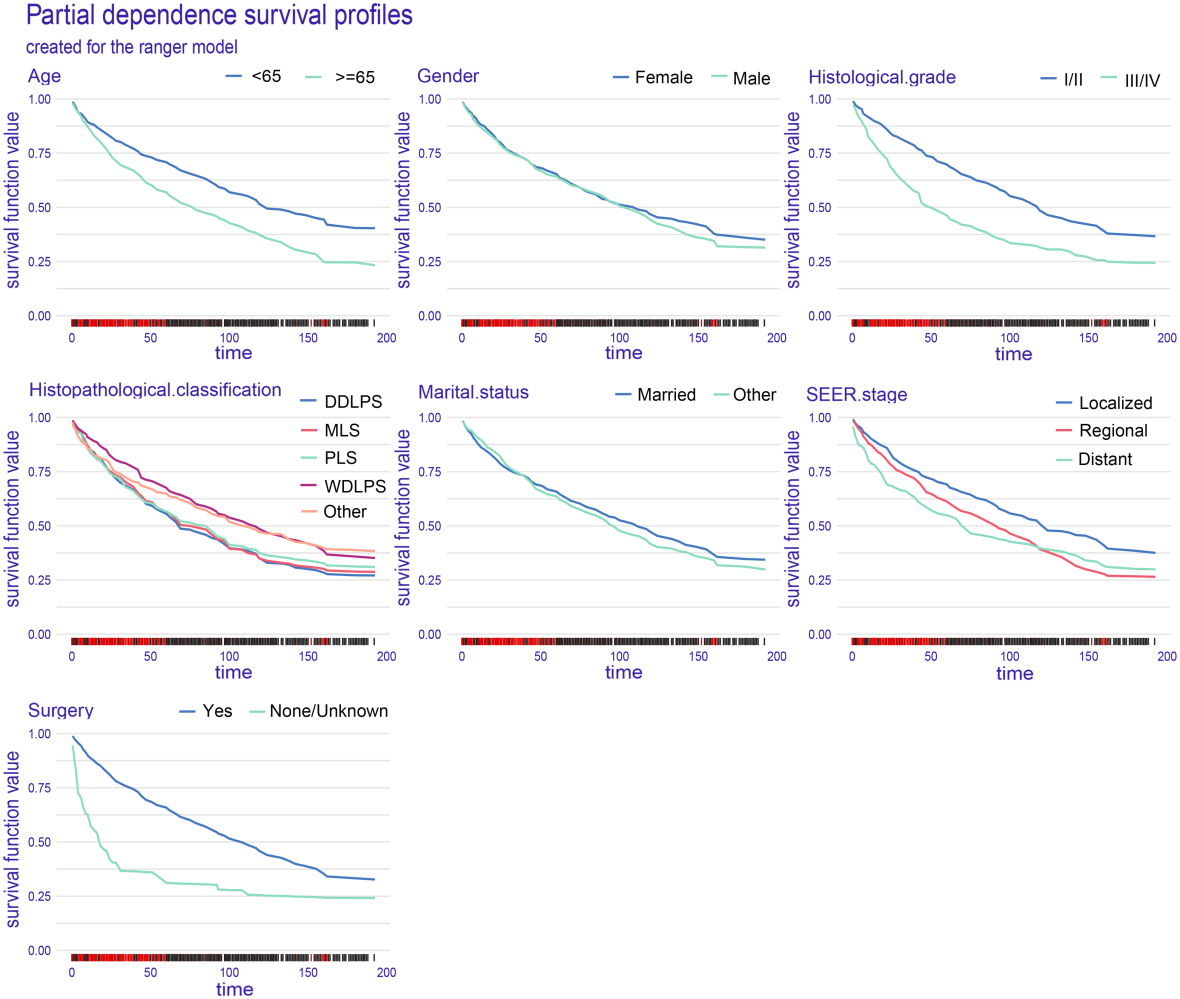
PDP can provide a global explanation for the ranger model. The survival function values of any covariates are depicted on the y‐axis. The larger the differences between levels of a factor, the greater the impact of the same factor on OS. A lower numerical value indicates a lower probability of survival.

Partial dependence survival profiles (PDP) can also provide a global explanation for the ranger model (Figure 9). PDP illustrates how the OS of the entire cohort changes with respect to the survival time when only a single determinant is altered while keeping all other determinants constant in the training dataset. If the plotted bands are thin and nearly overlapping, it indicates that the overall predictions remain similar regardless of the values of these variables. On the other hand, variables with wide bands imply that even slight changes in their values, such as surgery, age, and histological grade, can lead to significant variations. Additionally, the survival function of nonsurgical patients declines at a faster rate compared to postoperative patients. Similarly, patients aged 65 and above, as well as those with histological grade of III/IV, experience a faster decline in survival function.

#### 3.4.3 Aggregated SurvSHAP values summary

**FIGURE 10:**
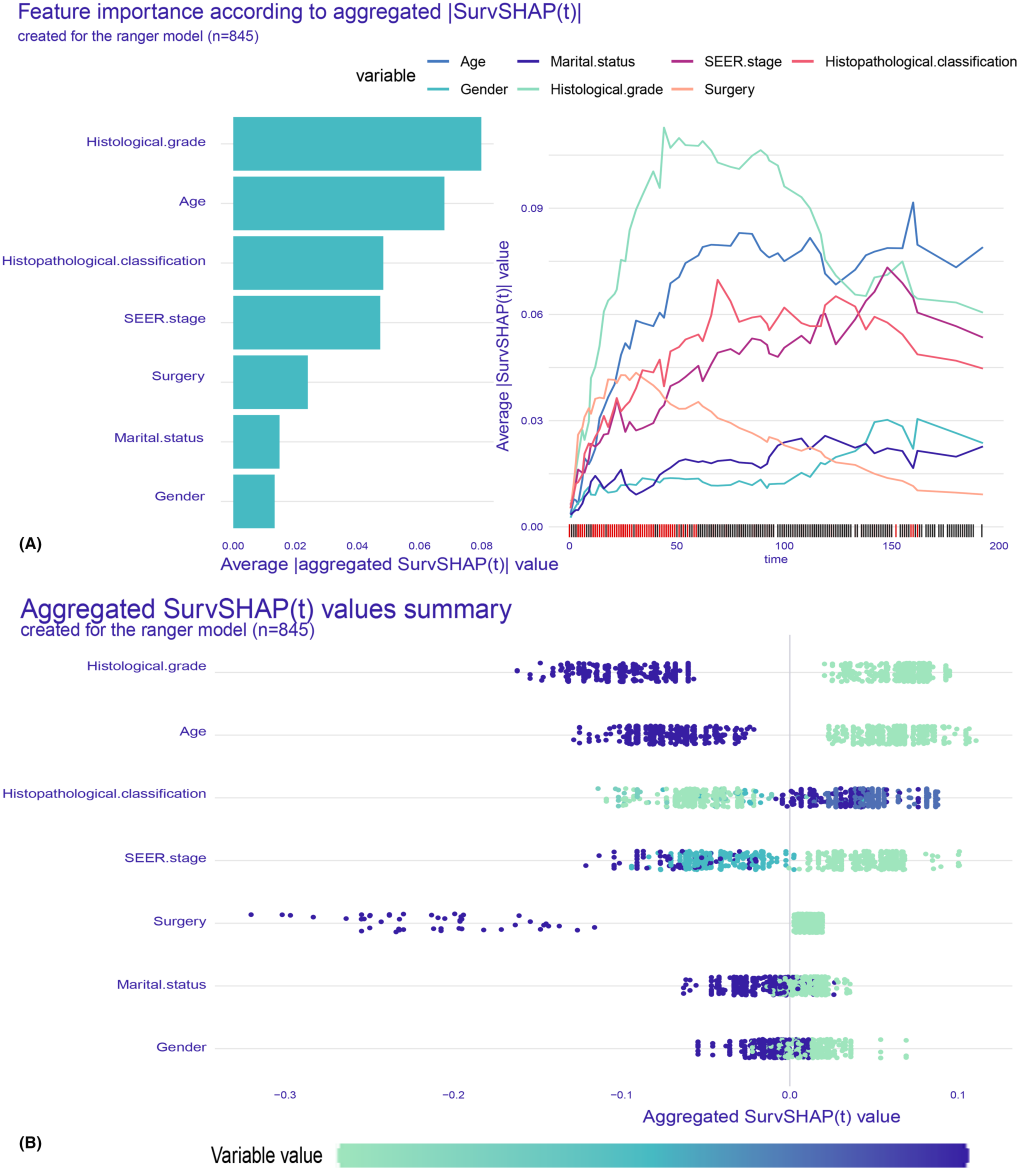
The SurvSHAP summary plot provides an overall interpretation of the global impact. (A) The length of the bar chart represents the overall significance of the variables, while the curve graph displays the cumulative importance of each variable. (B) Each point on the bee swarm plot represents a specific feature of a particular patient. The y‐coordinate of the point is determined by the feature it represents, while the x‐coordinate is determined by its impact on the model output. The color of the point indicates its value from high to low, as shown by the color bar below. The features on the *y*‐axis are sorted according to their significance.

SurvSHAP(t) expands the concept of SHAP to encompass a diverse range of models operating on survival data.^30^ We have calculated and illustrated the SurvSHAP summary plot of the ranger prediction model, which comprises seven features ranked according to their influence on OS. The left panel of Figure 10A illustrated the overall significance of variables, while the right panel displayed the temporal variability of each variable's importance, calculated as the average absolute SHAP value across all observation points. In the bee swarm plot, model variables were arranged in descending order of importance (Figure 10B). Higher SHAP values for features signified a greater impact on OS. Purple represented high feature values, blue represented feature values near the overall average, and green represented low feature values. Histological grade exhibited the highest level of impact, followed by factors such as age, histopathological classification, SEER stage, and surgery. For comparative purposes, please refer to Figure S7, which presents the bee swarm plot of the CoxPH model.

### 3.5 Local explanation

#### 3.5.1 SurvSHAP(t) plot per single patient

**FIGURE 11:**
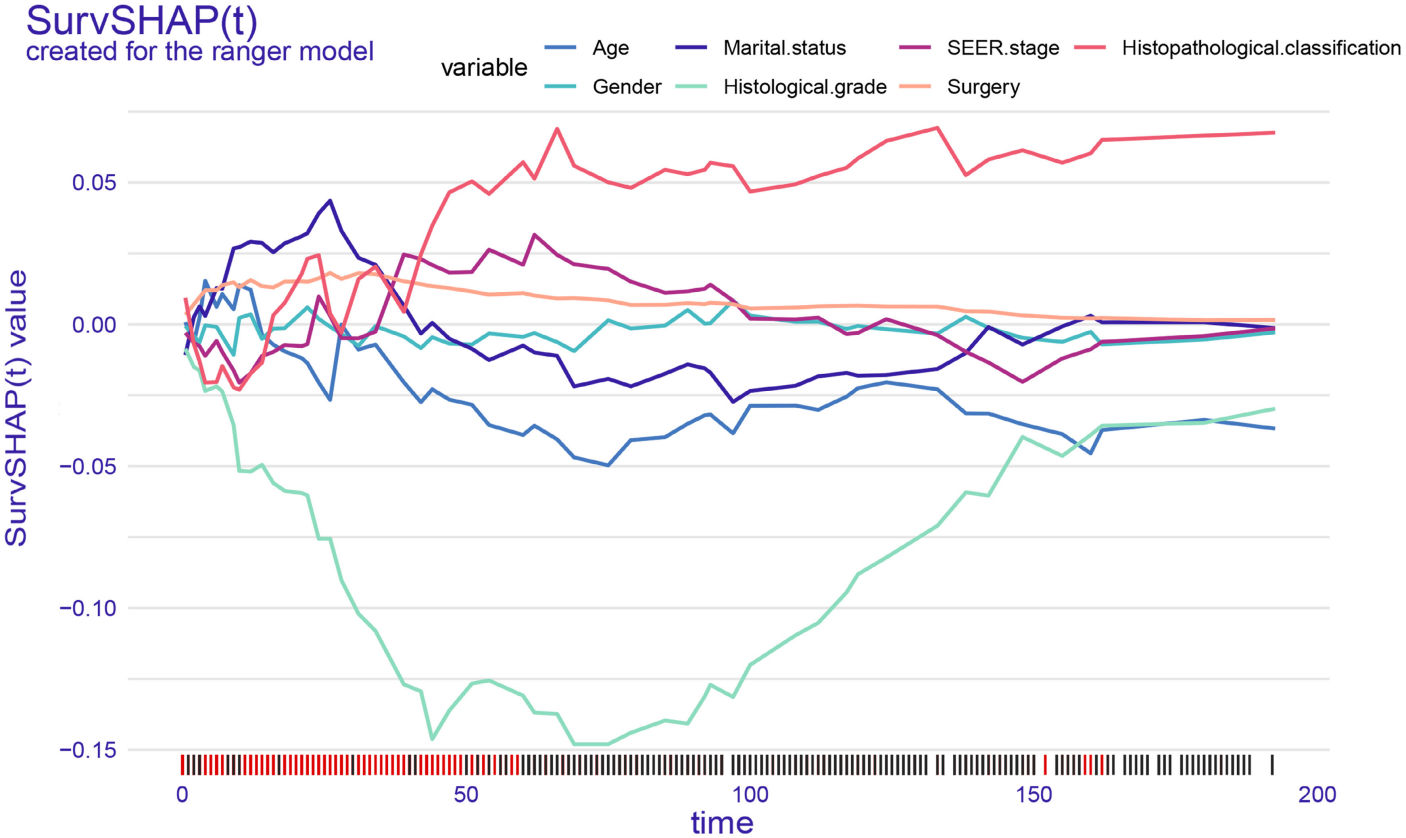
SurvSHAP (t) plot for a single patient. Plotting the positive SurvSHAP (t) values of each covariate on the *y*‐axis is associated with longer OS, while negative values are associated with shorter OS.

The SurvSHAP (t) plot allows for the assessment of time‐dependent survival contributions of risk factors on OS in each selected individual patient (Figure 11). In this case, we observed a divorced male patient who was older than 65 years old and had a history of RLPS surgery, poorly differentiated tumor, and histopathological classification as round cell. The tumor of this patient was classified as regional according to SEER staging. The results of the analysis indicated that the values of the histopathological classification variable increased the survival probability in this individual. Conversely, the value of the histological grade and age variables decreased the survival probability. Therefore, the main influencing factors on OS determined by SurvSHAP (t) were age, poorly differentiated tumor, and round cell.

#### 3.5.2 SurvLIME plot per single patient

**FIGURE 12:**
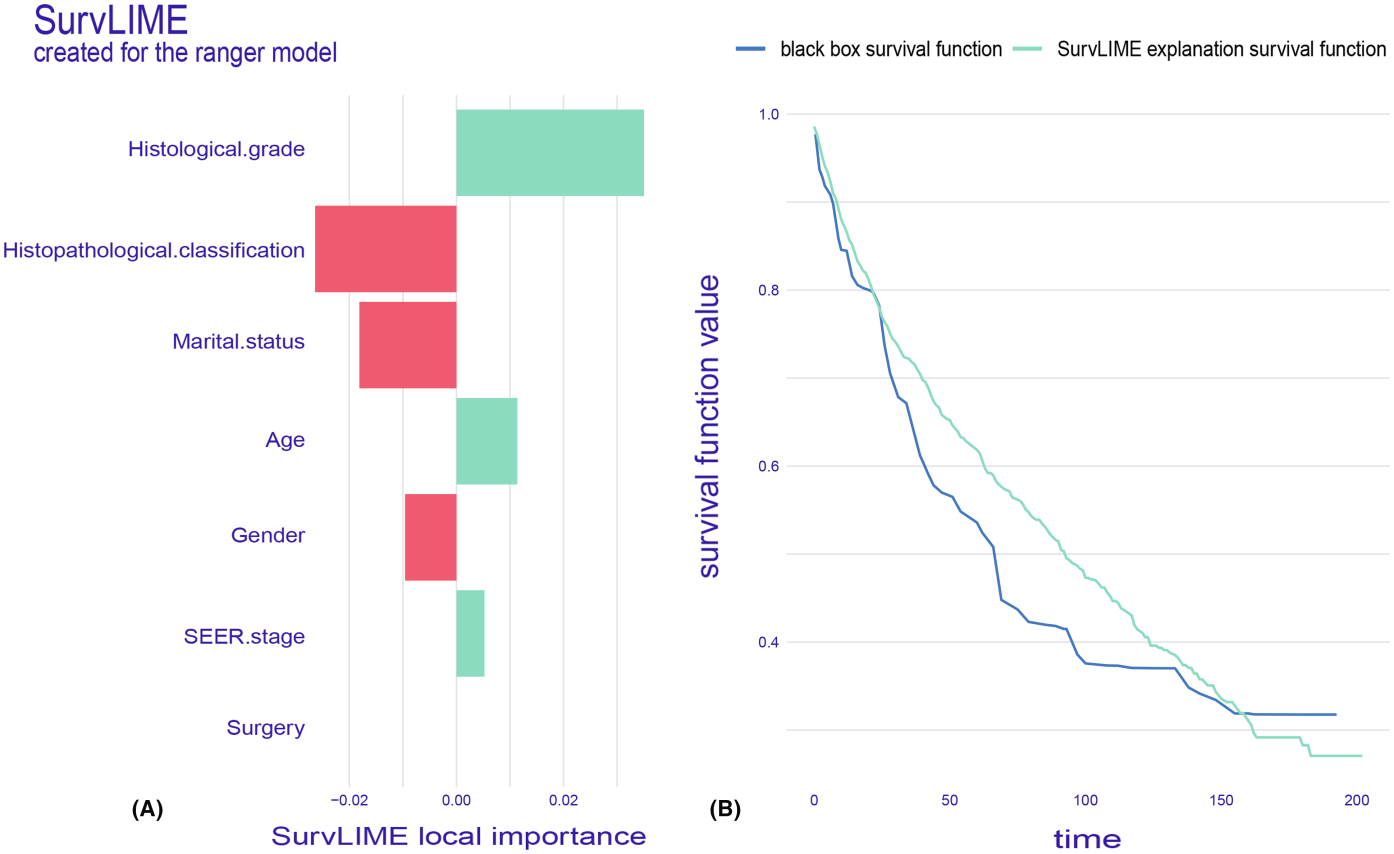
SurvLIME plot for a single patient. (A) The most significant variables were displayed, where higher values of local importance indicated lower survival chances. (B) The right figure depicted the predictions of the black box model and the identified alternative model.

The SurvLIME algorithm aims to rank within a given individual's feature set while calculating local interpretability considering time and space. The SurvLIME plotting method is closely related to SurvSHAP (t), both of which can detect the most influential survival prediction factors for a single selected patient (Figure 12). It can also generate a set of neighborhoods and obtain predictions for these neighborhoods. Finally, a local interpreter is fitted to minimize the distance between the predictions provided by the black box model and those provided by the local interpreter.^33^ The closer they are, the more accurate the survival estimation. In our local interpretation for the same patient mentioned above, the analysis results showed that factors such as histological grade, age, and SEER stage were associated with low survival. Conversely, factors such as histopathological classification, marital status, and gender were associated with high survival. In conclusion, poorly differentiated tumors, round cells for histopathological classification, as well as divorce status and age were identified as the most critical influencing factors for OS, which was consistent with the results of SurvSHAP (t). It is worth noting that the model on the right showed that the two curves were close, indicating accurate survival estimation.

## 4 DISCUSSION

RLPS is a rare tumor located in the retroperitoneal area. The prognosis of patients with RLPS has traditionally been associated with surgical treatment and histological subtypes of the tumor.^34^, ^35^ Previous studies have evaluated risk factors affecting the prognosis of RLPS patients, mainly through case reports and small sample studies.^34^, ^36^ Li et al. evaluated the prognosis of RLPS patients by using information from the SEER database and employing a Cox regression model.^37^ However, the model only included five prognostic risk factors, and the AUC for 1, 3, and 5‐year OS were all below 0.80. Considering the unclear prognostic value of clinical and pathological characteristics in RLPS patients and the limited accuracy of previous prognostic models, we constructed multiple machine learning models and compared their performance to identify the optimal model with superior survival prognostic capabilities.

ML algorithms have demonstrated significant advantages in cancer management, as evidenced by numerous studies conducted in recent years that have employed ML algorithms to construct models for predicting specific clinical endpoints. These studies indicate that ML algorithms are gradually being incorporated into clinical practice. Qu et al. employed six ML algorithms to construct a prognostic model for occult breast cancer, which revealed that the logistic regression (LR) model performed the best, accurately predicting the OS of patients with occult breast cancer.^38^ Similarly, Ryu et al. utilized ML algorithms like RSF to predict the OS of patients with spinal cord ependymoma.^39^ These studies also utilized data from the SEER database. In our study, six prediction models were constructed, including five models that utilized machine learning algorithms. By selecting seven conventional clinical and treatment features, we aimed to predict OS of RLPS patients. To the best of our knowledge, this is the first study to utilize multiple machine learning algorithms for predicting the survival status of RLPS patients. These models demonstrated good predictive capability based on a substantial amount of patient data from the SEER database. The performance of all models was evaluated through internal and external validation, with the ranger model performing the best. The explainability analysis of the ranger model indicated that patient age, as well as histological grade and histopathological classification related to the tumor, were the most important variables.

This study identified several independent factors associated with poor prognostic factors, including age over 65, male gender, unmarried statuses, absences of surgery, tumor histological grade III/IV, SEER stage of local or distant, and histopathological classification of DDLPS. According to previous study, a research report on liposarcoma indicated that age was an independent prognostic factor.^40^ In our study, patients with RLPS over 65 years old had poorer OS, and age was found to be a relatively significant risk factor in the explainability analysis. Hoffman et al. suggested that the prognosis of male liposarcoma patients was worse than that of females, possibly due to the impact of RASSF1A mutation (Ras‐association domain family member 1) in MLS.^41^, ^42^ A previous study has shown that married patients had an earlier disease stage at diagnosis and better survival outcomes compared to divorced, separated, or widowed RLPS patients.^43^ Similarly, multiple studies on bladder cancer and prostate carcinoma have also found that married patients had significantly better prognoses than unmarried patients.^44^, ^45^ Sánchez‐Hidalgo et al. conducted a retrospective analysis on the overall survival (OS) of 35 patients with RLPS and found that histological subtype, surgical margin, histological grade, and multifocality were factors associated with recurrence. Additionally, early recurrence was correlated with decreased OS (HR = 4.05; 95% CI: 1.27–12.96; *p* = 0.018).^46^ Regarding tumor stage, Bonvalot et al. performed a multivariate analysis on 290 patients without gross residual disease or tumor rupture and found that tumor stage was the only prognostic factor retained (*p* < 0.001).^47^ Our study also showed that patients with distant and regional SEER staging had worse OS compared to those with local SEER staging. Among the histopathological classification, DDLPS had a worse prognosis compared to other types.^48^, ^49^ Previous studies have shown that DDLPS has a four‐fold higher overall mortality risks than WDLPS, with WDLPS having the lowest risk among all subtypes.^50^ These findings were consistent with our study results.

In terms of treatment, patients who underwent surgical resection have a significantly better prognosis compared to those who did not undergo surgical resection. However, previous studies had primarily focused on the impact of surgical resection completeness on prognosis, and complete resection is an important predictor of prolonged OS.^51^, ^52^ Currently, there is no consensus on the efficacy of chemotherapy and radiotherapy for RLPS, and there are limited studies on patients who have undergone adjuvant therapy, particularly chemotherapy. Radiotherapy and chemotherapy are generally ineffective for the majority of RLPS cases, with a chemotherapy response rate of less than 10%.^53^ Our study did not consider these factors as influencing factors. However, previous studies have shown that adding preoperative radiotherapy to extensive surgical resection of RLPS may improve local control rates, and MLS is sensitive to radiation.^54^, ^55^ Therefore, new adjuvant therapies for RLPS may offer a broader prospect in the future and provide hope for improving patient OS.

Unlike previous ML research, our study not only provides global explanations for the optimal ranger model but also utilizes local explanations to explore the model's predictions for individual selected observations, resulting in distinct visualization outcomes. We can intuitively estimate the impact of any single determinant on the OS of each patient, although the results may demonstrate varying variations. Therefore, this study offers a more comprehensive and direct explanation of the survival model for RLPS, whether it is for the entire study population or specific patients.

Our study still has certain limitations. First, due to the retrospective design of the study, the possibility of selection bias cannot be avoided. Second, RLPS is a rare disease, resulting in a relatively small sample size. Despite the excellent predictive performance demonstrated by the ML models, future validation is necessary with larger datasets. Third, the SEER database does not include important molecular diagnostic information, such as PD‐1 and Ki‐67. Additionally, due to racial differences, the SEER database may not always be applicable to Asian populations. Lastly, the information about RLPS adjuvant therapy is incomplete, and further research is needed to better understand the impact of treatment plans on prognosis.

## 5 CONCLUSIONS

In this study, six machine learning models were developed to predict the OS of RLPS patients. The performance of these models was satisfactory, with the ranger model demonstrating superior predictive ability. We conducted an explainability analysis to provide both global and local explanations of the model, which highlighted the accuracy and intuitiveness of machine learning prediction models. Furthermore, machine learning models are capable of discerning intricate relationships among variables. We utilize these models to analyze relevant information from past similar patients, thereby predicting outcomes for newly considered patients. Despite the clinical diseases' complexity and heterogeneity, explainable machine learning models offer valuable and crucial references for clinical physicians to make informed decisions in advance. Additionally, they contribute to the development of prognostic methods for other types of cancer.

## AUTHOR CONTRIBUTIONS

**Maoyu Wang:** Methodology (equal); software (equal); writing – original draft (equal). **Zhizhou Li:** Data curation (equal); formal analysis (equal); writing – original draft (equal). **Shuxiong Zeng:** Conceptualization (equal); methodology (equal); project administration (equal); software (equal). **Ziwei Wang:** Data curation (equal); resources (equal). **Yidie Ying:** Data curation (equal); investigation (equal); resources (equal). **Wei He:** Conceptualization (equal); data curation (equal). **Zhensheng Zhang:** Project administration (equal); writing – review and editing (equal). **Huiqing Wang:** Project administration (equal); writing – review and editing (equal). **Chuanliang Xu:** Project administration (equal); writing – review and editing (equal).

## FUNDING INFORMATION

This study was funded by the National Natural Science Foundation of China (81772720, 81972391, and 82172871) and the “Voyaging Talents” Fund (2021008149), and the “234 Disciplines Peak Climbing Plan” Fund (2020YXK019) of the Naval Medical University.

## CONFLICT OF INTEREST STATEMENT

The authors have no relevant financial or nonfinancial interests to disclose.

## ETHICAL APPROVAL STATEMENT

We obtained authorization to access the research data file within the SEER program from the National Cancer Institute in the United States. The SEER data are publicly available and have been de‐identified. The study received approval from the Committee on Ethics of Medicine at Shanghai Changhai Hospital (No. CHEC2019‐142).

## Supporting information


Figure S1.
Figure S2.
Figure S3.
Figure S4.
Figure S5.
Figure S6.
Figure S7.

Table S1:

## Data Availability

The datasets generated and/or analyzed during the current study are available from the corresponding author upon reasonable request.
