# Supplementary material for: Explainable machine learning predicts survival of retroperitoneal liposarcoma: A study based on the SEER database and external validation in China
Source: Cancer Med. 2024 Jun 7;13(11):e7324. doi: 10.1002/cam4.7324 (PMC11157677; doi:10.1002/cam4.7324)
Supplement: Supplementary file 1 — Figure S1. Figure S2. Figure S3. Figure S4. Figure S5. Figure S6. Figure S7. [file CAM4-13-e7324-s001.docx]

**Explainable machine learning predict survival of** **retroperitoneal liposarcoma: a study based on the SEER database and external validation in China**

Supplementary figure 1 Model performance for the validation set was displayed in the form of bar plots.

Supplementary figure 2 Model performance for the validation set was displayed as a line chart that changes over time.

Supplementary figure 3 Model performance for the external validation set was displayed in the form of bar plots.

Supplementary figure 4 Model performance for the external validation set was displayed as a line chart that changes over time.

Supplementary figure 5 The Cox-Snell residual plots were displayed for all models in the validation dataset.

Supplementary figure 6 The Cox-Snell residual plots were displayed for all models in the external validation dataset.

Supplementary figure 7 The SurvSHAP summary plot provides an overall interpretation of the global impact for the CoxPH model.


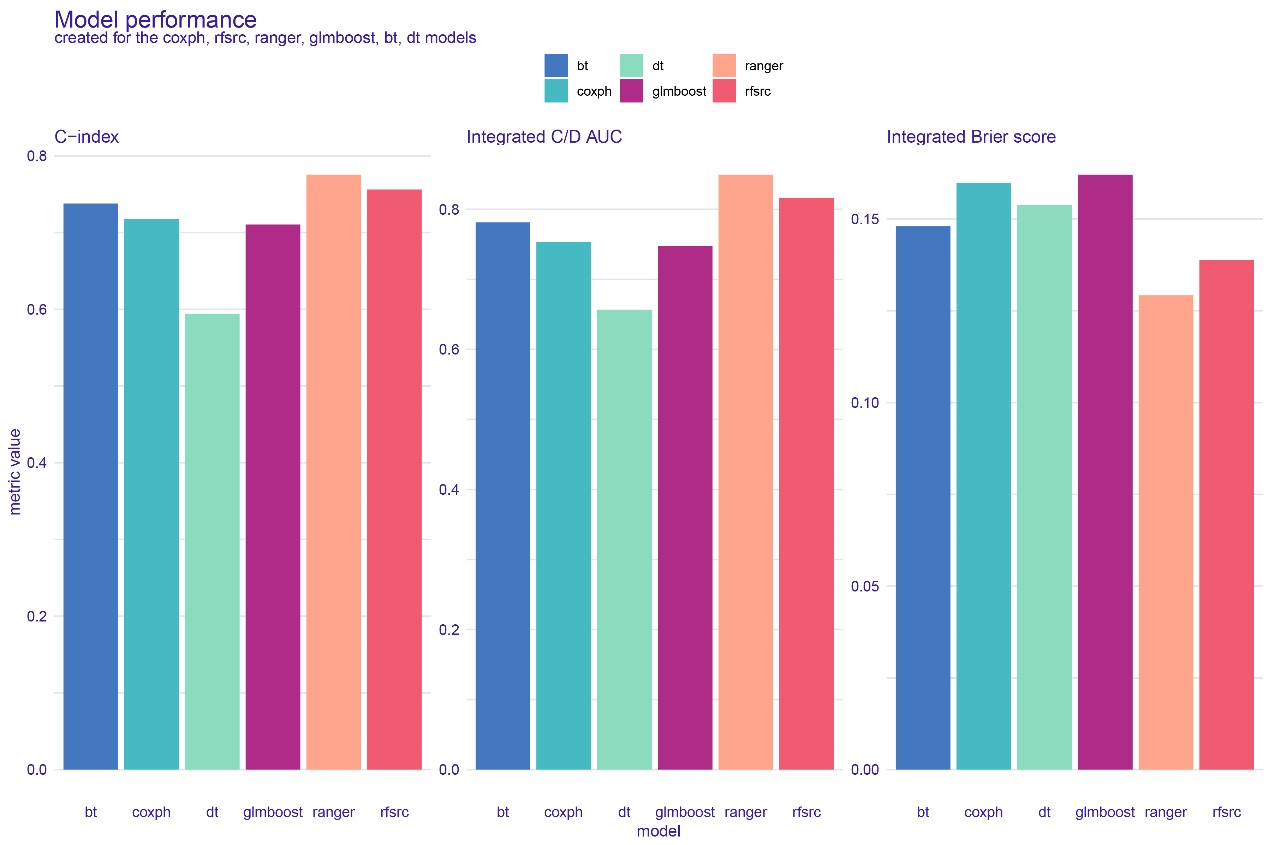


Supplementary figure 1 Model performance for the validation set was displayed in the form of bar plots.


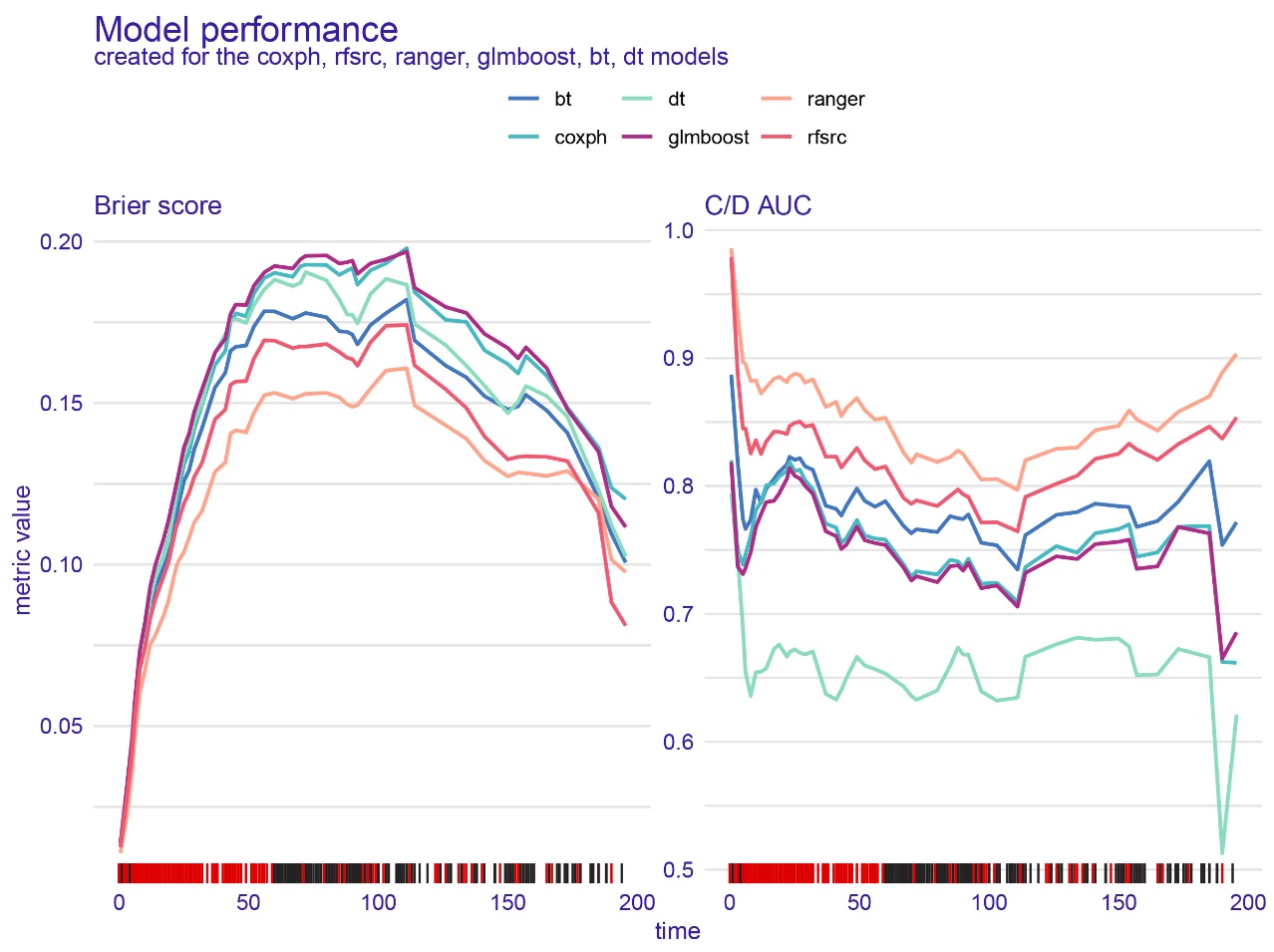


Supplementary figure 2 Model performance for the validation set was displayed as a line chart that changes over time.


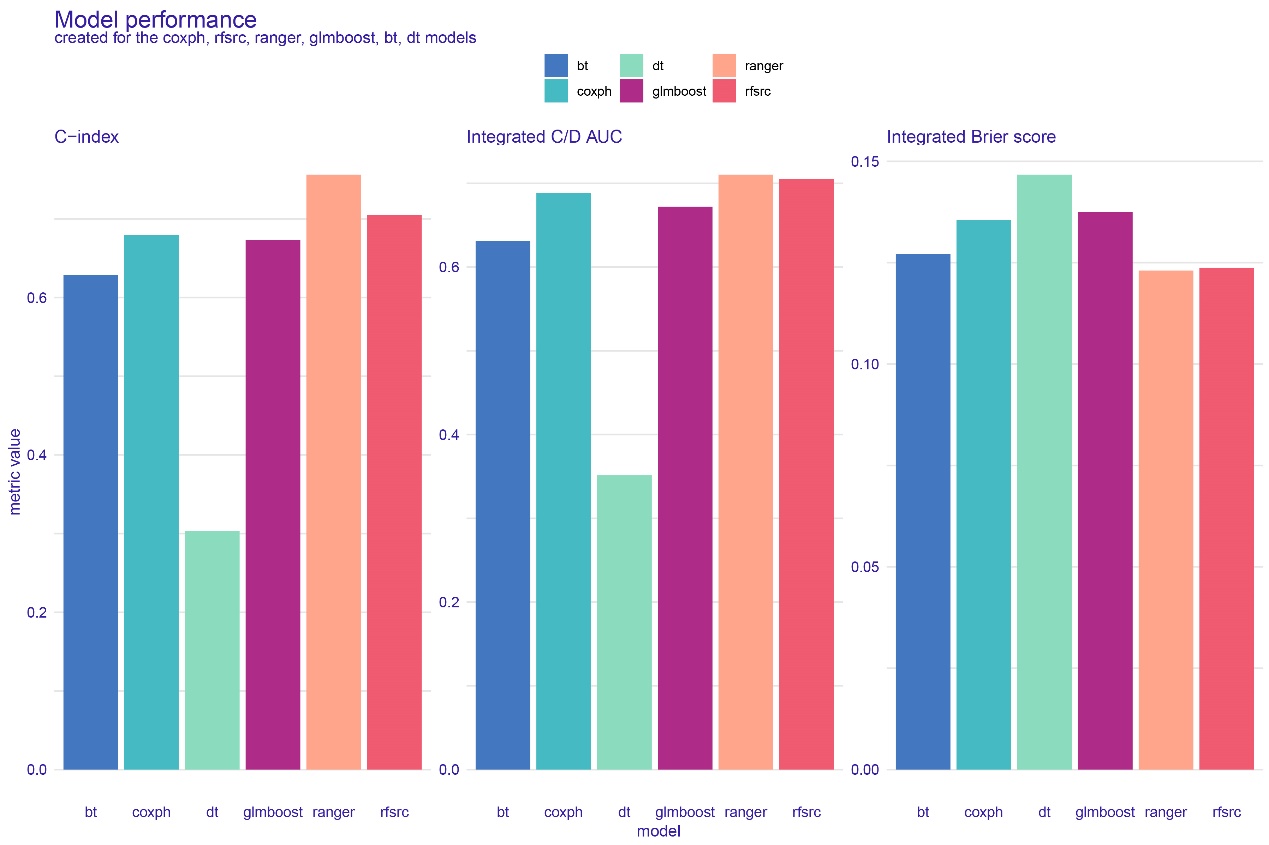


Supplementary figure 3 Model performance for the external validation set was displayed in the form of bar plots.


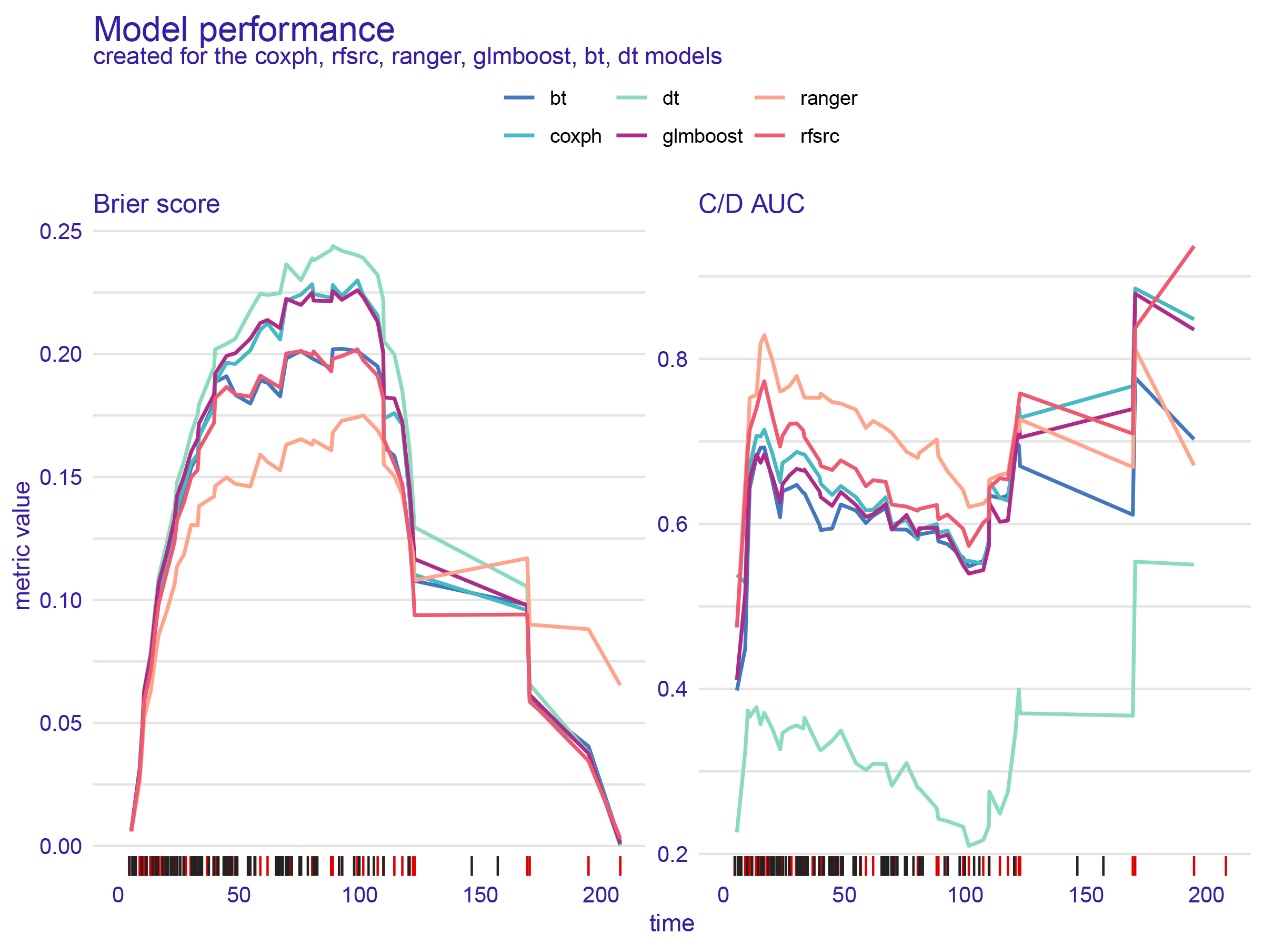


Supplementary figure 4 Model performance for the external validation set was displayed as a line chart that changes over time.


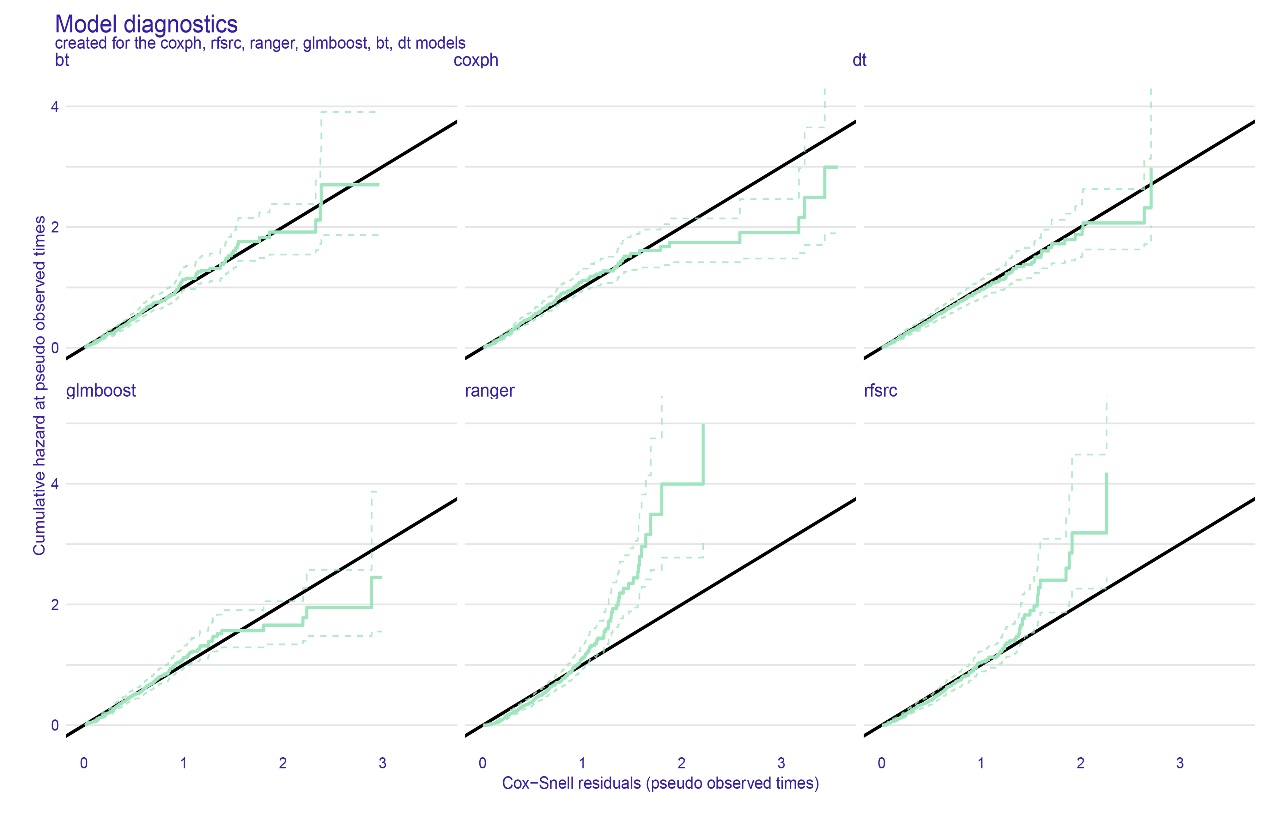


Supplementary figure 5 The Cox-Snell residual plots were displayed for all models in the validation dataset.


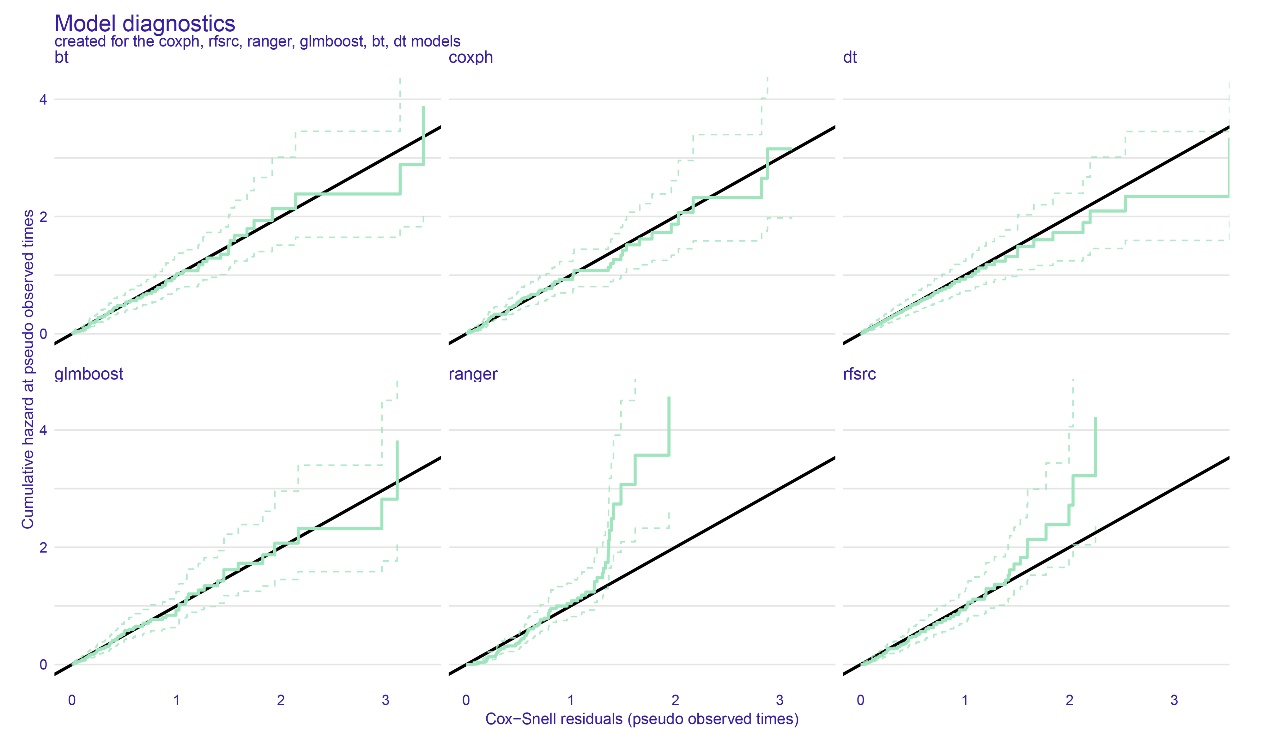


Supplementary figure 6 The Cox-Snell residual plots were displayed for all models in the external validation dataset.


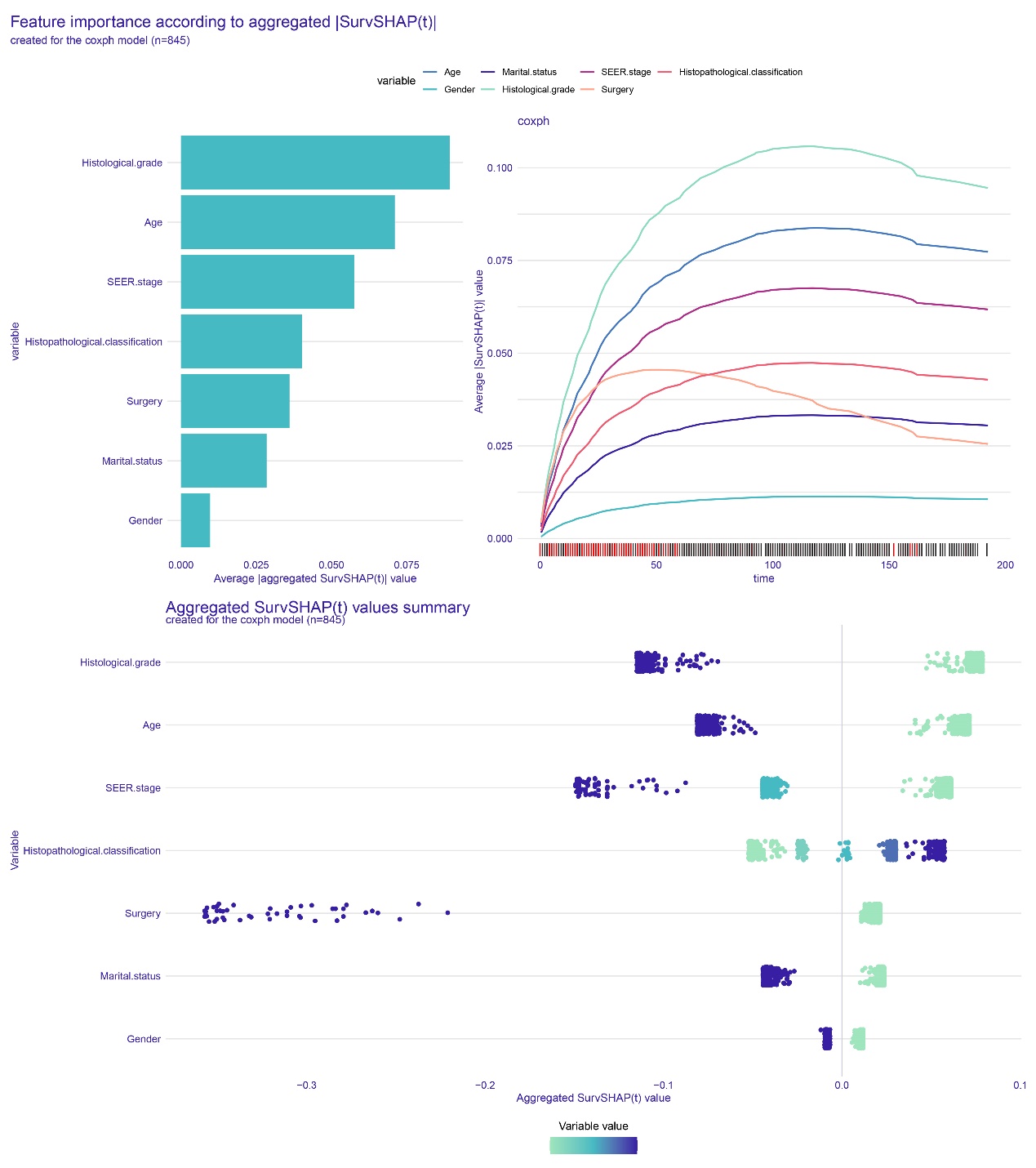


Supplementary figure 7 The SurvSHAP summary plot provides an overall interpretation of the global impact for the CoxPH model.
